# Supplementary material for: Experiences and perceptions of respectful maternity care among mothers during childbirth in health facilities of Eastern province of Rwanda: An appreciative inquiry
Source: PLoS One. 2025 Jan 17;20(1):e0315541. doi: 10.1371/journal.pone.0315541 (PMC11741570; doi:10.1371/journal.pone.0315541)
Supplement: Attachment — Submitted filename: REVIEW Rwanda.pdf [file pone.0315541.s004.pdf]

Experiences and perceptions of respectful maternity care among mothers during childbirth in health facilities of Eastern province of Rwanda: An Appreciative Inquiry"

The research primarily explores how mothers in Rwanda's Eastern Province perceive and appreciate their childbirth experiences, specifically focusing on Respectful Maternity Care (RMC). This question is highly relevant, as understanding positive RMC experiences can offer valuable insights into effective practices and culturally appropriate solutions, particularly in resource-limited settings. The focus on positive experiences through Appreciative Inquiry (AI) adds a unique dimension to the existing literature, which often emphasizes negative aspects like disrespect and abuse.

Ethical Concerns: The study appears to have addressed ethical considerations appropriately. The authors obtained necessary approvals from relevant institutional review boards and ensured informed consent from participants. No ethical concerns are readily apparent from the information provided.

Originality and Contribution: While RMC research exists in Rwanda, this study distinguishes itself by employing the AI approach to highlight positive childbirth experiences. This shift in perspective offers a fresh lens to identify sustainable RMC practices. The study's contribution lies in showcasing successful RMC implementation in Rwanda, potentially inspiring similar approaches in other resource-constrained contexts.

Writing Quality and Clarity: The paper is generally well-written, with clear language and logical organization. The flow of ideas is smooth, making it easy to follow the authors' arguments. However, there is room for improvement in conciseness, particularly in the introduction and discussion sections, where some statements could be streamlined.

Introduction:

Example 1: The following sentence could be condensed while retaining its core message: ….. "Integrating this culture of respect into the journey of childbirth is essential to enhance the rights of women and newborns and the general well-being of the population."

Suggestion: "Integrating respect into childbirth practices is crucial for upholding maternal and neonatal rights and overall well-being."

Example 2: This sentence could be made more concise by eliminating redundancy: "Studies argue that disrespect and abuse (D&A) can impose long-term psychological damage and emotional trauma by decreasing women's confidence and self-esteem [7,9]."

Suggestion; "Disrespect and abuse during childbirth can cause long-term psychological harm and diminish women's confidence and self-esteem [7,9]."

Methods: Provide a clearer explanation of how the Appreciative Inquiry (AI) approach was operationalized in your interviews and data analysis. This will enhance the transparency and replicability of your study.

Conclusions and Evidence Alignment: The authors highlight the positive RMC experiences reported by mothers, emphasizing aspects like compassionate care, autonomy, and timely service provision. These findings are well-supported by participant quotes and align with the study's aim. However, the conclusion could be strengthened by explicitly linking the findings to the broader context of RMC in Rwanda and globally. For instance,

“This study emphasizes the importance of maintaining and promoting RMC to provide women with care centred around their needs and a positive childbirth experience……

Suggestion: Currently, the conclusion primarily summarizes the study's findings and offers recommendations. To strengthen it, the authors could discuss how their findings relate to existing RMC initiatives in Rwanda, such as the training programs mentioned earlier in the paper. Additionally, they could explore how their findings compare to global trends in RMC research and advocacy. This would help situate the study within a larger conversation and highlight its potential implications for policy and practice beyond the local context.

Credibility of Claims: The claims made in the study are generally credible, supported by qualitative data from in-depth interviews with mothers. The use of the AI approach, while innovative, might introduce some bias towards positive experiences. Including data on negative experiences or challenges faced, even within the AI framework, could enhance the credibility and balance of the findings.

Major Flaws

Limited discussion of potential bias: The authors acknowledge the potential for desirability bias due to interviews being conducted at the hospital exit.

“The other limitation was that the study was conducted at the hospital exit time when the mothers were still on the hospital premises; this can lead to desirability bias. To minimize this concern, participants were assured of the complete confidentiality of their information, and interviews were conducted in a private room”

However, *a more in-depth discussion of this bias and its potential impact on the findings is warranted*. that a more in-depth discussion of this bias and its potential influence or impact on the findings enrich this article

Underutilization of literature in the discussion: The discussion could be enriched by drawing more explicit connections between the study's findings and existing literature on RMC, both in Rwanda and globally. This would strengthen the study's contribution to the field.

These findings align with global evidence on RMC, highlighting the importance of emotional support, positive attitudes of healthcare providers, and effective communication during labour and childbirth [4,39,21,40,24]."
"Participants suggested explaining to the community the service provided in maternity, similar to the interventional study in Kenya, which suggested using open maternity days to engage the community in maternity services [22]."
"Participants highlighted interventions such as HCP's training, supervision, and mentorship for new graduates as key factors in sustaining and improving RMC. These findings align with previous interventional studies conducted in the field [22,46,48,49] that have demonstrated the effectiveness of such interventions in RMC promotion."

While the instances above reference other literature, the discussion could explore deeper into comparing and contrasting the study's findings with existing research, both globally and specifically within the Rwandan context. This would help to clarify the study's unique contribution and its implications for future research and practice
